# Human Papillomavirus Concordance Between Parents and Their Newborn Offspring: Results From the Finnish Family Human Papillomavirus Study

**DOI:** 10.1093/infdis/jiad330

**Published:** 2023-08-10

**Authors:** Nelli T Suominen, Tiina H Luukkaala, Claudie Laprise, Marjut A Haataja, Seija E Grénman, Stina M Syrjänen, Karolina Louvanto

**Affiliations:** Department of Obstetrics and Gynecology, Turku University Hospital, University of Turku, Turku, Finland; Department of Obstetrics and Gynecology, Vaasa Central Hospital, Vaasa, Finland; Research, Development and Innovation Center, Tampere University Hospital, Tampere, Finland; Health Sciences, Faculty of Social Sciences, Tampere University, Tampere, Finland; Faculty of Dental Medicine and Oral Health Sciences, McGill University, Montreal, Quebec, Canada; Department of Obstetrics and Gynecology, Turku University Hospital, University of Turku, Turku, Finland; Department of Obstetrics and Gynecology, Turku University Hospital, University of Turku, Turku, Finland; Department of Oral Pathology and Radiology, University of Turku, Turku, Finland; Department of Pathology, Turku University Hospital, Turku, Finland; Department of Oral Pathology and Radiology, University of Turku, Turku, Finland; Department of Obstetrics and Gynaecology, Faculty of Medicine and Health Technology, Tampere University, Tampere, Finland; Department of Obstetrics and Gynaecology, Tampere University Hospital, Tampere, Finland

**Keywords:** human papillomavirus, HPV, father, perinatal, vertical transmission

## Abstract

**Background:**

The knowledge on vertical human papillomavirus (HPV) transmission is limited. We aimed to determine whether HPV transmission from parents to their offspring occurs before or during birth.

**Methods:**

Altogether, 321 mothers, 134 fathers, and their 321 newborn offspring from the Finnish Family HPV study cohort were included. Parents’ genital and oral brush samples and semen samples were collected for HPV testing at baseline (36 weeks of pregnancy). Oral, genital, and umbilical samples from the newborn and placenta samples were collected for HPV testing immediately after delivery. HPV risk for the newborn was calculated from the mother's and father's HPV status by using logistic regression analyses.

**Results:**

Concordances between mothers’ and their newborns’ HPV genotype at any site were statistically significant with HPV-6, -16, -18, -31, and -56; odds ratios (ORs) ranged from 3.41 (95% confidence interval [CI], 1.80–6.48) for HPV-16 to 634 (95% CI, 28.5–14 087) for HPV-31. Father–newborn HPV concordance was statistically significant with HPV-6 and HPV-31 (ORs, 4.89 [95% CI, 1.09–21.9] and 65.0 [95% CI, 2.92–1448], respectively).

**Conclusions:**

The genotype-specific HPV concordance between parents and their newborn is suggestive for vertical HPV transmission. However, transmission from the father to the newborn remains more uncertain.

## Teaser

Knowledge on vertical human papillomavirus (HPV) transmission from parents to their offspring is limited. We showed genotype-specific HPV concordance between mother–newborn and father–newborn pairs, suggestive for vertical HPV transmission occurrence before and/or during birth. However, father-to-newborn transmission remains more uncertain.

Evidence from the past 30 years supports the view that mucosal human papillomaviruses (HPVs) have multiple modes of transmission including both sexual and nonsexual transmission [1–5]. Among children, 1 route of transmission is vertical transmission from the mother or possibly from the father to the offspring [4, 6]. Perinatal transmission from mother to newborn is considered to result mainly from a close contact of infected birth canal during delivery [7], although HPV has also been detected in newborns born by cesarean delivery [8, 9]. Other possible HPV transmission modes from the mother to their offspring include intrauterine transmission as HPV has been detected in amniotic fluid [10, 11], placenta [10, 12–14], cord blood [10, 12, 14], and aborted products of conception [15].

Fathers’ role in prenatal HPV transmission remains unclear, and literature on the topic is scant. HPV has been widely detected in semen samples [16], suggesting possibility for periconceptual transmission (transmission at time around fertilization) from father to embryo [7]. One study has shown that in vitro artificially HPV infected sperm can be transmitted to the oocyte and that HPV genes can be actively transcribed by the penetrated oocyte [17].

To shed more light on the HPV transmission at prenatal period and at birth, our objective was to describe HPV genotype distribution among mothers and/or fathers and HPV concordance with their newborns using data from the Finnish Family HPV study.

## METHODS

### Finnish Family HPV Study

The Finnish Family HPV Study (FFHPV) is a prospective cohort study that was conducted at Turku University Hospital and University of Turku, Finland. Originally a total of 329 families with mothers, fathers, and their newborn offspring were recruited in the third trimester of pregnancy between 1998 and 2002. They were followed up with regular visits (including HPV testing in every visit and questionnaire at baseline and end of follow-up) for 6 years to elucidate the natural history of HPV infection between family members as previously described [18–20]. Participants’ earlier history of anogenital or oral lesions or warts did not affect study recruitment. History of possible HPV-related lesions was asked in detail in the baseline questionnaire of the study.

The study was performed in line with the principles of the Declaration of Helsinki. The study protocol and its amendment (numbers 3/1998, 2/2006, and 45/180/2010) were approved by the Ethics Committee of Turku University Hospital. Written informed consent to participate was obtained from all adult participants. Written informed consent for children's participation was obtained from both parents of each child.

### Sample Collection

The sample collection is described in detail in previous publications of the FFHPV study [12, 19, 20]. In brief, scraping samples for HPV testing from newborns, mothers, and fathers were collected using a cytobrush (MedScand, Malmö, Sweden). Mothers’ oral and cervical samples collected at baseline (36 weeks of pregnancy) were included in the present study. From the father, urethral, oral, and semen samples collected at baseline (36 weeks of pregnancy of their spouses) were also included; fathers’ urethral sample was defined as fathers’ genital sample. Oral and genital scraping samples were collected from newborns immediately after birth as well as placental and umbilical cord blood samples [12]. From the placenta, 2 representative samples covering all tissue layers were taken. Umbilical venous cord blood sample was taken while the placenta was still in situ. Oral samples from all participants were collected with the cytobrush from the buccal mucosa of both cheeks and from the upper and lower vestibular area. Genital brush samples were taken from the labia/prepuce and scrotum of the newborn, the cervical mucosa of the mother, and the distal part of the urethral mucosa of the father using a cytobrush. Cervical samples were placed in phosphate-buffered saline with 100 µg of gentamycin, and all other brush samples were placed in 70% ethanol, as described in detail elsewhere [20]. Semen collection and analyses were done by the guidelines of the Nordic Association for Andrology. Semen samples were taken into a plastic container by masturbation using gloves after at least 2 days of abstinence. If taken at home, the sample was transferred to the laboratory within 2 hours after ejaculation. Samples were centrifuged in a Sorval MC12V (Zurich, Switzerland) at 3500 rpm for 15 minutes. Seminal plasma and semen cells were stored separately, first at −20°C and afterward at −70°C.

### HPV Detection and Genotyping

During DNA extraction, contamination was carefully monitored by simultaneous DNA extraction from cultured human fibroblasts or HPV-negative immortalized human gingival keratinocytes, which served as negative controls for contamination during DNA extraction. Only 8 study samples were processed at the same time. For each set of 8 samples, 1 fibroblast-negative control and 1 HPV-16–positive control (Siha cell lines, human cervical epithelial carcinoma, HTB-35, American Type Culture Collection) were used.

HPV amplification was done by nested polymerase chain reaction (PCR) (MY09/MY11 external primers and GP05^+^/bioGP06^+^ internal primers) [18]. Contamination during HPV amplification was carefully controlled. In addition to the negative and positive controls described above, we also included 1 no-DNA sample for each set of 8 study samples to be processed for HPV amplification. DNA extraction, master mix for PCR, and addition of target DNA in the reaction mixture were all done in separate rooms that were exchanged regularly in the MediCity Research Laboratory, Faculty of Medicine, University of Turku, Finland. In these research rooms, neither any clinical samples nor HPV-positive cell lines were used.

HPV genotyping was done using a Luminex-based Multimetrix kit (Progen Biotechnik GmbH, Heidelberg, Germany), which identifies 24 different low-risk (LR) and high-risk (HR) HPV genotypes (LR-HPV: genotypes 6, 11, 42, 43, and 44; HR-HPV: genotypes 16, 18, 26, 31, 33, 35, 39, 45, 51, 52, 53, 56, 58, 59, 66, 68, 70, 73, and 82) as described earlier [21]. All HPV-16–positive samples were retested from the original samples using nested PCR and the Luminex-based assay for HPV-16 genotyping to rule out contamination during HPV amplification as these 2 methods shared only the DNA extracted from the original sample. Importantly, the samples collected from the family members were never analyzed at the same time including DNA extraction or HPV amplification. In addition, samples were stored in separate boxes.

### Statistical Analyses

From the original FFHPV study, we included 321 mothers, 134 fathers, and 321 of their newborn offspring comprising 321 mother–newborn and 134 father–newborn pairs. All included mothers and fathers had an HPV genotyping result available from the oral and/or genital mucosa before the birth of their offspring. Missing HPV data of fathers were included and modeled as unknown group. When HPV prevalence at any anatomic site was calculated, the person counted as HPV positive if his/her HPV sample was positive at least at 1 anatomic site. The distribution of HPV genotypes among family members was described as numbers with proportions (%) of HPV-positive newborns, mothers, and fathers.

The genotype-specific HPV concordance was defined when both parent and newborn tested positive for the specific HPV genotype. Vertical transmission rate was calculated for mother–newborn and father–newborn pairs. The proportion of mothers and fathers having concordant HPV genotype with her/his offspring was calculated by each HPV genotype as well. Associations of type-specific HPV presence between mother–newborn and father–newborn pairs were determined by using univariable logistic regression analysis and reporting results by odds ratios (ORs) with 95% confidence intervals (CIs). The reference category for the specific HPV genotype positivity was the negativity for that HPV genotype. Concordance between mothers’/fathers’ and newborns’ genotype-specific HPV status was also assessed by using Cohen kappa (κ) method. The following benchmark scale for interpreting the κ statistics by Landis and Koch [22] was used to describe the degree of agreement (HPV concordance) between mother–newborn and father–newborn pairs: <0.00, poor; 0.00–0.20, slight; 0.21–0.40, fair; 0.41–0.60, moderate; 0.61–0.80, substantial; and 0.81–1.00, almost perfect degree of agreement.

HPV genotypes were grouped to LR- and HR-HPV groups. Associations of newborns’ LR- and HR-HPV presence with mothers’ and fathers’ corresponding LR- and HR-HPV presence were determined by using univariable and another-parent adjusted (mothers’ HPV adjusted by fathers’ HPV and vice versa) multinomial (LR- and HR-HPV types) logistic regression analyses and reporting results by ORs and another-parent adjusted odds ratios (aORs) with 95% CI. The reference category for LR- and HR-HPV was HPV negative. IBM SPSS Statistics version 26.0 for Windows software (IBM SPSS Inc, Chicago, Illinois) was used for statistical analyses. All tests were 2-sided and *P* values<.05 were considered statistically significant.

## RESULTS

Mean age of mothers was 25.5 years (standard deviation [SD], 3.4; range, 18–46) and mean age of fathers was 28.8 years (SD, 5.0; range, 19–46). The frequencies of different HPV genotypes in newborns’ (n = 321), mothers’ (n = 321), and fathers’ (n = 134) samples are shown in Table 1. Detailed HPV type distributions by the different anatomic sites are shown in Supplementary Table 1. Among newborns, mothers, and fathers, HPV prevalence at any anatomic site was 31.2% (n = 100), 31.2% (n = 100), and 45.5% (n = 61), respectively. HPV-16 was the most common HPV genotype identified in all groups (newborns, mothers, fathers), followed by HPV-6. Among newborns, HPV-16 and HPV-6 were found in 15.9% (n = 51) and 4.7% (n = 15) of samples, respectively. HPV-16 accounted for 20.9% (n = 67) of mothers’ and 23.9% (n = 32) of fathers’ HPV infections, whereas HPV-6 was found in 4.7% (n = 15) of mothers’ samples and in 9.7% (n = 13) of fathers’ samples. Newborns’ oral HPV prevalence was 23.0% (n = 74) and genital HPV prevalence was 10.0% (n = 32). For the HPV genotype distribution, multiple-type infections were sorted as individual HPV genotypes. Two or more different HPV genotypes detected in the same sample was defined as a multiple infection, which accounted for 5.9% (n = 19) of newborns’ HPV infections, 10.3% (n = 33) of mothers’ HPV infections, and 18.2% (n = 24) of fathers’ HPV infections.

**Table 1.** Distribution of Human Papillomavirus (HPV) Genotypes Among the Family Members of the Finnish Family HPV Study

HPV Genotype	Newborn (n = 321)		Mother (n = 321)		Father (n = 134)	
	No.	(%)	No.	(%)	No.	(%)
HPV negative	221	(68.8)	221	(68.8)	73	(54.5)
Any HPV	100	(31.2)	100	(31.2)	61	(45.5)
6	15	(4.7)	15	(4.7)	13	(9.7)
11	1	(0.3)	6	(1.9)	5	(3.7)
16	51	(15.9)	67	(20.9)	32	(23.9)
18	7	(2.2)	9	(2.8)	4	(3.0)
31	3	(0.9)	3	(0.9)	3	(2.2)
33	13	(4.0)	3	(0.9)	10	(7.5)
35	0	…	2	(0.6)	0	…
39	4	(1.2)	1	(0.3)	0	…
42	0	…	4	(1.2)	0	…
43	0	…	2	(0.6)	2	(1.5)
45	2	(0.6)	5	(1.6)	1	(0.7)
51	0	…	2	(0.6)	2	(1.5)
52	0	…	1	(0.3)	0	…
53	1	(0.3)	0	…	4	(3.0)
56	5	(1.6)	6	(1.9)	2	(1.5)
58	2	(0.6)	8	(2.5)	0	…
59	5	(1.6)	4	(1.2)	1	(0.7)
66	9	(2.8)	9	(2.8)	2	(1.5)
68	1	(0.3)	0	…	0	…
70	2	(0.6)	2	(0.6)	5	(3.7)
73	1	(0.3)	0	…	0	…
82	1	(0.3)	1	(0.3)	5	(3.7)
Multiple-type HPV	19	(5.9)	33	(10.3)	24	(18.2)

The proportions (%) of HPV genotypes in newborns’ (oral/genital/umbilical cord blood/placenta) samples collected at birth, and mothers’ (oral/genital) and fathers’ (oral/genital/semen) samples collected at baseline (ie, before birth) are shown. Multiple-type HPV infections were sorted as individual HPV genotypes. No. indicates number of HPV types found. HPV types missing for father: n = 187.

Abbreviation: HPV, human papillomavirus.

Rate of vertical transmission with any HPV type was 37.0% (37/100) and 35.1% (33/94) from mothers’ any anatomic site to newborns’ any anatomic site, and mothers’ genital site to newborns’ any anatomic site, respectively. From fathers’ any anatomic site to newborns’ any anatomic site, vertical transmission rate was shown to be 19.7% (12/61). Type-specific HPV prevalence was then further utilized to calculate newborns’ risk for infection by each HPV genotype separately (Table 2). At first, we looked for HPV presence at all anatomic sites pooled together and evaluated genotype-specific HPV concordances between mother–newborn pairs and father–newborn pairs. Mother–newborn genotype-specific HPV concordances were statistically significant with HPV-6, -16, -18, -31, and -56, with ORs ranging from 3.41 (95% CI, 1.80–6.48) for HPV-16 to 634 (95% CI, 28.5–14 087) for HPV-31. Father–newborn HPV concordances were statistically significant with HPV-6 (OR, 4.89 [95% CI, 1.09–21.9]) and HPV-31 (OR, 65.0 [95% CI, 2.92–1448]). A total of 60% (9/15), 31% (21/67), 22% (2/9), 67% (2/3), and 50% (3/6) of HPV-6–, HPV-16–, HPV-18–, HPV-31–, and HPV-56–positive mothers, respectively, had concordant HPV genotype with their newborn. Of HPV-6– and HPV-31–positive fathers, 23% (3/13) and 33% (1/3), respectively, had concordant HPV genotype with their newborn. HPV concordances between newborns’ and mothers’ genotype-specific HPV status was substantial with HPV-31 (κ = 0.66) and moderate both with HPV-6 (κ = 0.58) and HPV-56 (κ = 0.54), as seen in Table 2.

**Table 2.** Associations of Type-Specific Human Papillomavirus (HPV) Presence and Degree of HPV Concordance Between Mother–Newborn and Father–Newborn Pairs Among Family Members of the Finnish Family HPV Study

HPV Type	Newborn and Mother (n = 321)				Newborn HPV due to Mother (n = 321)				Newborn and Father (n = 134)				Newborn HPV due to Father (n = 134)			
	Both Negative	Child Positive	Mother Positive	Both Positive	%	κ	OR	(95% CI)	Both Negative	Child Positive	Father Positive	Both Positive	%	κ	OR	(95% CI)
6	300	6	6	9	60	**0.58**	**75.0**	**(20.2–278)**	114	7	10	3	23	0.19	**4.89**	**(1.09–21.9)**
11	314	1	6	0					128	1	4	0				
16	224	30	46	21	31	0.21	**3.41**	**(1.80–6.48)**	86	16	24	8	25	0.10	1.79	(.67–4.69)
18	307	5	7	2	22	0.23	**17.5**	**(2.89–106)**	130	0	4	0				
31	317	1	1	2	67	**0.66**	**634**	**(28.5–14 087)**	130	1	2	1	33	0.39	**65.0**	**(2.92–1448)**
33	305	13	3	0					121	3	10	0				
39	317	3	0	1					133	1	0	0				
45	314	2	5	0					132	1	1	0				
53	320	1	0	0					129	1	4	0				
56	313	2	3	3	50	**0.54**	**157**	**(18.8–1304)**	132	0	2	0				
58	313	0	6	2					133	1	0	0				
59	312	5	4	0					133	0	1	0				
66	303	9	9	0					128	4	2	0				
68	320	1	0	0					136	0	0	0				
70	317	2	2	0					129	0	5	0				
73	320	1	0	0					136	0	0	0				
82	319	1	1	0					128	1	5	0				

% indicates proportions of mothers/fathers with concordant HPV genotype with their offspring. Cohen kappa (κ) shows the degree of HPV concordance between mothers/fathers and their newborns for each HPV genotype.

The following benchmark scale describes the degree of agreement (HPV concordance): <0.00, poor; 0.00–0.20, slight; 0.21–0.40, fair; 0.41–0.60, moderate; 0.61–0.80, substantial; 0.81–1.00, almost perfect degree of agreement.

Association of type-specific HPV presence between mothers/fathers and their newborns was determined by using unadjusted logistic regression analysis. Results are shown by ORs with 95% CIs. The reference category for specific HPV genotype positivity was the negativity for that HPV genotype. Moderate or higher degree of agreement (κ ≥ 0.41) and statistically significant ORs (*P* < .05) are shown in bold. Empty cells indicate results that cannot be computed due to zero frequencies.

Abbreviations: CI, confidence interval; HPV, human papillomavirus; OR, odds ratio.

Newborns had a clearly increased risk for 5 different HPV genotype in our first evaluations, which led us to assess which anatomic site would have the most impact on these results. For these evaluations, we grouped the HPV genotypes by LR- and HR-HPV. In Supplementary Table 2 we show the associations of the presence of newborns’ LR- and HR-HPV at any anatomic site with their mother’s or father’s corresponding LR- and HR-HPV at specific anatomic sites. Mothers’ oral HR-HPV infection appeared to increase the risk of newborns’ any anatomic site HR-HPV infection both in univariable and father-adjusted models. Furthermore, mothers’ multiple-site (HPV detected at least 2 different anatomic site) LR- and HR-HPV infections were associated with newborns’ any anatomic site LR- and HR-HPV presence; these associations stayed statistically significant also in the father-adjusted model.

The impact on newborns’ site-specific HPV prevalence was further evaluated. First, we investigated the associations of the presence of newborns’ oral LR- and HR-HPV with the mother’s or father’s corresponding LR- and HR-HPV at specific anatomic sites (oral, genital, semen, multiple sites) as seen in Table 3. When the impact of parents’ oral HPV on newborns’ oral HPV infection were analyzed, both LR- and HR-HPV detected at the mother's oral site was related to newborns’ oral LR- and HR-HPV, whereas at the father's oral site, only HR-HPV was associated with newborns’ oral HR-HPV. These associations stayed also statistically significant in the another parent-adjusted model. The presence of fathers’ genital LR-HPV was related to newborns’ oral LR-HPV infection, and the association was seen also in the mother-adjusted model. There was no association between mothers’ genital LR- or HR-HPV infection and newborns’ oral LR- or HR-HPV infection.

**Table 3.** Association of Newborns’ Oral Low-Risk and High-Risk Human Papillomavirus (HPV) Presence With Their Mothers’ and Fathers’ Corresponding Low-Risk and High-Risk HPV Presence at Specific Anatomic Sites Among Family Members of the Finnish Family HPV Study

Parent and Site	Newborns’ Oral HPV Prevalence at Birth			Univariable Model		Adjusted Model^a^	
	HPV Negative (n = 247)	LR-HPV^b^ (n = 12)	HR-HPV^b^ (n = 62)	LR-HPV^b^	HR-HPV^b^	LR-HPV^b^	HR-HPV^b^
	No. (%)	No. (%)	No. (%)	OR (95% CI)	OR (95% CI)	aOR (95% CI)	aOR (95% CI)
Mother							
Negative	184 (74.5)	5 (41.7)	32 (51.6)	1.00	1.00	1.00	1.00
Oral	23 (9.3)	3 (25.0)	17 (27.4)	**4.80 (1.08–21.4)**	**4.25 (2.05–8.83)**	**7.12 (1.44–35.1)**	**4.87 (2.29–10.4)**
Genital	36 (14.6)	2 (16.7)	7 (11.3)	2.04 (.38–11.0)	1.12 (.46–2.73)	2.76 (.48–15.7)	1.16 (.47–2.90)
Multiple sites	4 (1.6)	2 (16.7)	6 (9.7)	**18.4 (2.71–126)**	**8.63 (2.30–32.3)**	**10.4 (1.30–82.9)**	**8.28 (2.12–32.3)**
Father							
Negative	60 (24.3)	3 (25.0)	10 (16.1)	1.00	1.00	1.00	1.00
Oral	7 (2.8)	1 (8.3)	7 (11.3)	2.86 (.26–31.3)	**6.00 (1.73–20.8)**	3.89 (.33–45.9)	**7.72 (2.10–28.3)**
Genital	9 (3.6)	3 (25.0)	3 (4.8)	**6.67 (1.16–38.2)**	2.00 (.46–8.68)	**7.93 (1.12–56.1)**	2.10 (.43–10.2)
Semen	12 (4.9)	0	2 (3.2)	…	1.00 (.19–5.15)	…	1.29 (.24–6.98)
Multiple sites	14 (5.7)	0	3 (4.8)	…	1.29 (.31–5.29)	…	1.34 (.30–5.87)
Unknown	145 (58.7)	5 (4.7)	37 (59.7)	0.69 (.16–2.98)	1.53 (.72–3.28)	0.72 (.17–3.29)	1.63 (.74–3.63)

Newborns’ (n = 321) oral HPV prevalence at birth is shown with their mothers’ and fathers’ baseline HPV status at specific anatomic sites. Associations of newborns' oral LR- and HR-HPV presence with their mothers' and fathers' corresponding LR- and HR-HPV presence at specific anatomic sites were calculated by using univariable and another-parent adjusted multinomial logistic regression analyses. Results are shown by ORs and another-parent aORs with 95% CIs. Reference category for HPV positive was HPV negative. Statistically significant results (*P* < .05) are shown in bold.

Abbreviations: aOR, another-parent adjusted odds ratio; CI, confidence interval; HPV, human papillomavirus; HR, high-risk; LR, low-risk; OR, odds ratio.

^a^Mothers' HPV adjusted by fathers' HPV and vice versa.

^b^LR-HPV genotypes: 6, 11, 42, 43, 44; HR-HPV genotypes: 16, 18, 26, 31, 33, 35, 39, 45, 51, 52, 53, 56, 58, 59, 66, 68, 70, 73, 82.

Last we evaluated the influence of mothers’ and fathers’ HPV status on newborns’ genital HPV infection as described in Table 4. Only mothers’ genital HR-HPV infection showed to be the significant risk factor for newborns’ genital HR-HPV infection, which strengthened in father-adjusted analysis.

**Table 4.** Association of Newborns' Genital Low-Risk and High-Risk Human Papillomavirus (HPV) Presence With Their Mothers' and Fathers' Corresponding Low-Risk and High-Risk HPV Presence at Specific Anatomic Sites Among the Family Members of the Finnish Family HPV Study

Parent and Sites	Newborns' Genital HPV Prevalence at Birth			Univariable Model		Adjusted Model^a^	
	HPV Negative (n = 289)	LR-HPV^b^ (n = 3)	HR-HPV^b^ (n = 29)	LR-HPV^b^	HR-HPV^b^	LR-HPV^b^	HR-HPV^b^
	No. (%)	No. (%)	No. (%)	OR (95% CI)	OR (95% CI)	aOR (95% CI)	aOR (95% CI)
Mother							
Negative	204 (70.6)	0	17 (58.6)	1.00	1.00	1.00	1.00
Oral	41 (14.2)	0	2 (6.9)		0.59 (.13–2.63)		0.63 (.14–2.87)
Genital	34 (11.8)	2 (66.7)	9 (31.0)		**3.18 (1.31–7.70)**		**3.34 (1.35–8.30)**
Multiple sites	10 (3.5)	1 (33.3)	1 (3.4)		1.20 (.14–9.94)		1.06 (.12–9.18)
Father							
Negative	66 (22.8)	0	7 (24.1)	1.00	1.00	1.00	1.00
Oral	13 (4.5)	1 (33.3)	1 (3.4)		0.73 (.08–6.40)		0.74 (.08–6.71)
Genital	12 (4.2)	1 (33.3)	2 (6.9)		1.57 (.29–8.50)		1.70 (.30–9.67)
Semen	11 (3.8)	0	3 (10.3)		2.57 (.58–11.5)		2.03 (.43–9.55)
Multiple sites	17 (5.9)	0	0				
Unknown	170 (58.8)	1 (33.3)	16 (55.2)		0.89 (.35–2.56)		0.87 (.34–2.24)

Newborns' (n = 321) genital HPV prevalence at birth is shown with their mothers' and fathers' baseline HPV status at specific anatomic sites. Associations of newborns' genital LR- and HR-HPV presence with their mothers' and fathers' corresponding LR- and HR-HPV presence at specific anatomic sites were calculated by using univariable and another-parent adjusted multinomial logistic regression analyses. Results are shown by ORs and another-parent aORs with 95% CIs. Reference category for HPV positive was HPV negative. Statistically significant results (*P* < .05) are shown in bold.

Abbreviations: aOR, another-parent adjusted odds ratio; CI, confidence interval; HPV, human papillomavirus; HR, high-risk; LR, low-risk; OR, odds ratio.

^a^Mothers' HPV adjusted by fathers' HPV and vice versa.

^b^LR-HPV genotypes: 6, 11, 42, 43, 44; HR-HPV genotypes: 16, 18, 26, 31, 33, 35, 39, 45, 51, 52, 53, 56, 58, 59, 66, 68, 70, 73, 82.

## DISCUSSION

We discovered that there is a genotype-specific HPV concordance between newborns and their mother and/or father, which indicates the likelihood of vertical HPV transmission between the parents and their newborn offspring. Furthermore, parents’ oral HR-HPV presence seems to impact more on the newborn's oral HR-HPV infection risk than parents’ genital HR-HPV presence. On the other hand, newborns’ genital HR-HPV risk was only predicted by the mother's HR-HPV presence before delivery.

According to 1 meta-analysis [23], vertical transmission solely from mothers (genital sample) to newborns (oral/genital sample) is estimated to be 6.5% with a wide range between 1.5% and 46.6%. Fewer studies have evaluated vertical transmission based on genotype-specific mother–newborn HPV concordance [8, 9, 24–30]. To date, only 1 meta-analysis [31] of type-specific intrauterine vertical transmission has been published, suggesting an intrauterine transmission rate of 4.9%, but the rate of transmission in selected studies varied between 0% and 46.7%. We observed a transmission rate of 37.0% (37/100) from mothers’ any anatomic site to newborns’ any anatomic site, and transmission rate of 35.1% (33/94) from mothers’ genital site to newborns’ any anatomic site.

A previous study [32] that investigated the co-occurrence of HPV-16/18 infection between 146 Polish parental couples and their newborns showed that mothers’ any anatomic site and genital and oral combined HPV-16/18 infection, as well as fathers’ oral HPV-16/18 infection, increased the risk of newborns’ oral HPV-16/18 infection at birth. These results are in line with our finding, as we showed that mothers’ and fathers’ oral HR-HPV infections increase the risk of newborns’ oral HR-HPV infection at birth. Another study [28] investigated the vertical genotype-specific HPV transmission from both parents to the newborns with a US population in Iowa; 574 mothers and newborns and 68 fathers participated in the study. In contradiction, only 1 mother–newborn pair and no father–newborn pairs showed HPV genotype-specific concordance, suggesting that vertical transmission from parents to newborn is rare (<1%) [28]. However, this study had a slightly different study setting from ours, as HPV samples from fathers’ oral site and newborns’ oral and genital sites were taken 65 hours after birth.

We observed an HPV prevalence of 31.2%, 23.0%, and 10.0% in newborns’ any anatomic, oral, and genital sites, respectively, which is higher than the previously reported prevalence among newborns [8, 24, 26, 28, 29, 33]. Two studies reported HPV prevalence of 22.4% (11/49) and 23.5% (55/233) at any anatomic site [8, 24]. Similar to our study, prevalence was based on at least 2 different anatomic sample sites. Moreover, several studies have reported oral prevalence of ≤4% [26, 28, 29]. The differences in HPV prevalence can be explained by the sensitivity of the methods used for HPV testing and differences of sample size as well as timing of sampling after birth. We used accurate nested PCR in HPV testing, and the possibility of false-positive results due to contamination was minimized by using a negative control in every eighth sample during isolation and PCR. We selected nested PCR for HPV testing from the very beginning as the collection of sufficient amounts of nucleated epithelial cells from newborns’ oral or genital mucosa is demanding and viral copies in these cells is estimated to be low.

HPV-16 was the most common HR-HPV type detected in all sites of mothers and newborns, which is consistent with other reports [8, 25, 26, 29, 30]. However, the possible consequences of early-life HR-HPV infection are still not known due to the lack of long-term follow-up studies. The second most detected HPV type in newborns was HPV-6, which is known to cause severe juvenile-onset recurrent respiratory papillomatosis [34]. Our results suggest that HPV genotypes 6, 16, 18, 31, and 56 appear to be transmitted from mother to the newborn. This result is in line with a previous study in which the most frequent HPV type occurring in both newborns’ nasopharyngeal aspirates and mothers’ cervical samples was HPV-16 (10 pairs) [9]. The concordant HPV types except HPV-56 found in our present study could be vaccinated against with 9-valent vaccine, which we should bear in mind when adjusting vaccination strategies (ie, optimal age to vaccinate children). As maternal HPV immunoglobulin G antibodies are transferred to offspring [35], a future mode to prevent perinatal HPV infection could be HPV vaccination of potential parents. On the other hand, debate continues regarding whether newborns’ HPV infections at birth and during the perinatal period represent a true infection or contamination of the mother's birth canal. Even though a majority of the HPV infections detected at birth are shown to clear during the first months of life [24, 26, 29, 36, 37], our recent study showed that oral HPV infection detected at birth can persist a mean duration of 20.6 months [37]. Moreover, we have showed seroconversion of HPV-6, -11, -16, and -18 antibodies among children born to HPV-seronegative mothers, which indicates that a child may create the immune response to HPV in early infancy [35]. This current study now strengthens the assumption that the mother may not be the only source of HPV in newborns’ prenatal HPV infection.

Our study has several strengths, which first include evaluation of genotype-specific HPV concordance between both parents and their offspring. We acknowledge that genotype-specific concordance between mothers’ or fathers’ HPV status and newborns’ HPV status at birth most likely represents vertical transmission, including possibility for intrauterine transmission. Second, as the majority of previous studies have focused on evaluation of vertical transmission only between the mother and the newborn, we also included fathers to elucidate the possible role of periconceptual transmission, which remains mostly unexplored. Interestingly, we showed HPV-6 and HPV-31 genotype-specific concordance between fathers’ baseline HPV status and newborns’ HPV status at birth, which suggests fathers’ possible role in periconceptual HPV transmission. Our final strength is our sampling performance as we determined HPV status on multiple anatomical sites including cord blood and placenta, which enabled us to observe accurately the signs of intrauterine transmission.

Some limitations should also be considered. Our cohort had a lower number of fathers (n = 134) than mothers (n = 321), due to fathers’ unwillingness to participate in the study. In addition, parental samples were collected only at 36 weeks of pregnancy. The most precise information of different modes of vertical transmission, including periconceptual transmission, could be better shown with multiple consecutive sampling of both parents from before fertilization to the end of pregnancy. Another limitation is that we could not stratify analysis by HPV genotype when evaluating in which anatomic site parental HPV positivity most affects the likelihood of newborns’ HPV infection. In our analysis, we had to group HPV types into LR and HR groups because HPV sample counts at different anatomic sites were too low for genotype-specific analyses; therefore, results should be validated further by larger studies in the future. Although we showed HPV concordance between fathers and newborns, it does not justify direct father-to-newborn transmission. Alternative to direct father-to-newborn transmission, the transmission to the newborn might have been vertical from the mother as mother and father are expected to share same HPV genotypes through sexual transmission. Even if the mother's HPV sample is negative, it might be a false-negative result. However, as we used accurate nested PCR for HPV detection, the possibility of false-negative results is low, particularly in mothers’ cervical samples in which viral load is supposed to be high. Another matter of debate is whether HPV DNA detected in newborns represents a true infection or passive contamination/passage, most importantly from the infected maternal genital tract or delivery room. In this study, transcriptionally active HPV was not examined, and thus the state of HPV infection as such could not be verified. In addition, we do not have information on the type-specific HPV variant via sequencing, which would provide more proof of the transmission between family members and should be taken into account in future studies.

In summary, we showed the HPV concordance between newborns’ HPV status at birth and their mother’s and father’s HPV status, the results of which suggest that parents’ HPV infection may play an important role in newborns’ susceptibility to gain perinatal HPV infection. However, the father's role as a transmitter remains more uncertain. The role of periconceptional transmission from the parent to the newborn and the consequences of perinatal HPV infection later in individuals’ life will need further investigations.

## Supplementary Data

Supplementary materials are available at *The Journal of Infectious Diseases* online. Consisting of data provided by the authors to benefit the reader, the posted materials are not copyedited and are the sole responsibility of the authors, so questions or comments should be addressed to the corresponding author.

## Supplementary Material

jiad330_Supplementary_Data

## Notes

***Acknowledgments***. The skillful technical assistance of Tatjana Pescova, Mariia Henttinen, and Keitlin Adel is gratefully acknowledged.

***Financial support***. This work was supported by the Academy of Finland (to S. M. S. and K. L.); Syöpäsäätiö (to S. M. S,); the Päivikki and Sakari Sohlberg Foundation (to S. M. S.); the Sigrid Jusélius Foundation (to K. L.); and the Finnish Medical Foundation (to K. L. and N. T. S.). Funding to pay the Open Access publication charges for this article was provided by University of Turku.
