# Supplementary material for: Human Papillomavirus Concordance Between Parents and Their Newborn Offspring: Results From the Finnish Family Human Papillomavirus Study
Source: J Infect Dis. 2023 Aug 10;229(2):448–56. doi: 10.1093/infdis/jiad330 (PMC10873173; doi:10.1093/infdis/jiad330)
Supplement: jiad330_Supplementary_Data [file jiad330_supplementary_data.zip › supplementary_table1_revised.docx]

**Supplementary Table 1** Distribution of HPV genotypes by the different anatomic sites among newborns and their mothers and fathers. Multiple-type infections were sorted out as individual HPV genotypes.

|  | HPV genotype positivity | | | | | | | | | | | | | | | | |
| --- | --- | --- | --- | --- | --- | --- | --- | --- | --- | --- | --- | --- | --- | --- | --- | --- | --- |
|  | 6 | 11 | 16 | 18 | 31 | 33 | 39 | 45 | 53 | 56 | 58 | 59 | 66 | 68 | 70 | 73 | 82 |
| **Newborns (N=321)^a^** |  |  |  |  |  |  |  |  |  |  |  |  |  |  |  |  |  |
| **Number of HPV+ newborns at any site** | **15** | **1** | **51** | **7** | **3** | **13** | **4** | **2** | **1** | **5** | **2** | **5** | **9** | **1** | **2** | **1** | **1** |
| Oral | 11 | 1 | 31 | 6 | 3 | 6 | 3 | 2 | 1 | 4 | 2 | 3 | 7 | 1 | 2 | 1 | 1 |
| Genital | 3 |  | 20 | 1 |  | 5 | 1 |  |  | 1 | 1 |  | 1 |  |  |  |  |
| Umbilical cord blood | 5 |  | 5 |  |  |  | 1 |  |  |  |  |  |  |  |  |  |  |
| Placenta | 5 |  | 7 |  |  |  |  |  |  |  |  |  |  |  |  | 1 |  |
|  |  |  |  |  |  |  |  |  |  |  |  |  |  |  |  |  |  |
| **Mothers (N=321)^b^** |  |  |  |  |  |  |  |  |  |  |  |  |  |  |  |  |  |
| **Number of HPV+ mothers at any site** | **15** | **6** | **67** | **9** | **3** | **3** | **1** | **5** | **0** | **6** | **8** | **4** | **9** | **0** | **2** | **0** | **1** |
| Oral | 9 | 1 | 39 | 3 |  |  | 1 |  |  | 1 | 5 |  | 3 |  |  |  |  |
| Genital | 6 | 5 | 31 | 6 | 3 | 3 |  | 5 |  | 5 | 3 | 3 | 6 |  | 2 |  | 1 |
|  |  |  |  |  |  |  |  |  |  |  |  |  |  |  |  |  |  |
| **Fathers (N=134)^c^** |  |  |  |  |  |  |  |  |  |  |  |  |  |  |  |  |  |
| **Number of HPV+ fathers at any site** | **13** | **5** | **32** | **4** | **3** | **10** | **0** | **1** | **4** | **2** | **0** | **1** | **2** | **0** | **5** | **0** | **5** |
| Oral | 1 | 2 | 12 | 1 | 1 | 4 |  |  |  |  |  |  |  |  | 1 |  | 5 |
| Genital | 8 | 1 | 13 |  | 1 | 5 |  |  | 3 | 2 |  |  | 1 |  | 3 |  |  |
| Semen | 8 | 2 | 12 | 3 | 1 | 5 |  | 1 | 1 |  |  | 1 | 2 |  | 1 |  |  |
|  |  |  |  |  |  |  |  |  |  |  |  |  |  |  |  |  |  |

**^a^Newborns (N=321):** HPV negative n=221; one HPV type found in 81 newborns; 2 different HPV types found in 15 newborns; 3 different HPV types found in 4 newborns

**^b^Mothers (N=321):** HPV negative n=221; one HPV type found in 67 mothers; 2 different HPV types found in 20 mothers; 3 different HPV types found in 9 mothers; 4 different HPV types found in 4 mothers

**^c^Fathers (N=134):** HPV negative n=73; one HPV type found in 37 fathers, 2 different HPV types found in 19 fathers, 3 different HPV types found in 4 fathers; 4 different HPV types found in 1 father
