# Supplementary material for: Human Papillomavirus Concordance Between Parents and Their Newborn Offspring: Results From the Finnish Family Human Papillomavirus Study
Source: J Infect Dis. 2023 Aug 10;229(2):448–56. doi: 10.1093/infdis/jiad330 (PMC10873173; doi:10.1093/infdis/jiad330)
Supplement: jiad330_Supplementary_Data [file jiad330_supplementary_data.zip › supplementary_table2_revised.docx]

**Supplementary Table 2** Associations of newborns’ LR- and HR-HPV presence at any anatomic site with their mothers’ and fathers’ corresponding LR- and HR-HPV presence at specific anatomic sites among the family members of the Finnish Family HPV Study

|  |  | | | | **Newborns’ HPV prevalence at birth** | | | | | |
| --- | --- | --- | --- | --- | --- | --- | --- | --- | --- | --- |
|  |  | | | | |  | **Univariable model** | | **Adjusted model^a^** | |
|  | HPV negative  n=221 | | LR-HPV^b^  n=13 | HR-HPV^b^  n=87 | |  | LR-HPV^b^ | HR-HPV^b^ | LR-HPV^b^ | HR-HPV^b^ |
|  | n (%) | | n (%) | n (%) | |  | OR (95% CI) | OR (95% CI) | aOR (95% CI) | aOR (95% CI) |
| **Mother** |  | |  |  | |  |  |  |  |  |
| Negative | 167 (74.9) | | 6 (46.2) | 48 (54.5) | |  | 1.00 | 1.00 | 1.00 | 1.00 |
| Oral | 21 (9.4) | | 3 (23.1) | 19 (21.6) | |  | 3.98 (0.92-17.1) | **3.15 (1.58-6.33)** | **4.75 (1.05-21.5)** | **3.57 (1.75-7.30)** |
| Genital | 30 (13.5) | | 2 (15.4) | 13 (14.8) | |  | 1.86 (0.36-9.63) | 1.51 (0.73-3.12) | 2.29 (0.43-12.2) | 1.55 (0.74-3.27) |
| Multiple site | 3 (1.3) | | 2 (15.4) | 7 (8.0) | |  | **18.6 (2.60-132.5)** | **8.12 (2.02-32.6)** | **13.2 (1.68-103.5)** | **7.52 (1.82-31.0)** |
| **Father** |  | |  |  | |  |  |  |  |  |
| Negative | 51 (22.9) | | 5 (38.5) | 17 (19.3) | |  | 1.00 | 1.00 | 1.00 | 1.00 |
| Oral | 6 (2.7) | | 1 (7.7) | 8 (9.1) | |  | 1.70 (0.17-17.1) | **4.00 (1.21-13.2)** | 1.99 (0.19-21.1) | **4.58 (1.34-15.6)** |
| Genital | 8 (3.6) | | 2 (15.4) | 5 (5.7) | |  | 2.55 (0.42-15.4) | 1.88 (0.54-6.51) | 2.37 (0.32-17.3) | 1.86 (0.49-6.99) |
| Semen | 9 (4.0) | | 0 | 5 (5.7) | |  | … | 1.67 (0.49-5.66) | … | 1.89 (0.54-6.63) |
| Multiple site | 14 (6.3) | | 0 | 3 (3.4) | |  | … | 0.64 (0.16-2.51) | … | 0.61 (0.15-2.49) |
|  | |  | | | | | | | | |

Newborns’ (N=321) HPV prevalence at birth are shown with their mothers’ and fathers’ baseline HPV status at specific anatomic sites. Associations of newborns’ LR- and HR-HPV presence at any anatomic site with their mothers’ and fathers’ corresponding LR- and HR-HPV presence at specific anatomic sites were calculated by using univariable- and another parent -adjusted multinomial logistic regression analyses. Results were shown by odds ratios (OR) and another-parent adjusted odds ratios (aOR) with 95% confidence intervals (CI). Reference category for HPV positive was HPV negative. Statistically significant results (p-value <0.05) are shown in bold.

Abbreviations: CI, confidence interval; OR, odds ratio; aOR, another-parent adjusted odds ratio; HPV, human papillomavirus; HR, high-risk LR, low-risk

^a^mothers’ HPV adjusted by fathers’ HPV and vice versa

^b^LR-HPV: 6, 11, 42, 43, 44; HR-HPV 16, 18, 26, 31, 33, 35, 39, 45, 51, 52, 53, 56, 58, 59, 66, 68, 70, 73, 82
